# Postmarket Surveillance of Medical Devices: A Comparison of Strategies in the US, EU, Japan, and China

**DOI:** 10.1371/journal.pmed.1001519

**Published:** 2013-09-24

**Authors:** Daniel B. Kramer, Yongtian T. Tan, Chiaki Sato, Aaron S. Kesselheim

**Affiliations:** 1 Harvard Medical School, Boston, Massachusetts, United States of America; 2 Cardiovascular Division, Beth Israel Deaconess Medical Center, Boston, Massachusetts, United States of America; 3 Policy Alternatives Research Institute, University of Tokyo, Toyko, Japan; 4 Division of Pharmacoepidemiology and Pharmacoeconomics, Department of Medicine, Brigham and Women's Hospital, Boston, Massachusetts, United States of America

## Abstract

Daniel Kramer and colleagues compare strategies for postmarket surveillance of medical devices and discuss ways to improve these systems.

*Please see later in the article for the Editors' Summary*

**Summary Points**

- We evaluated strategies for postmarket surveillance of medical devices in the United States, European Union, Japan, and China.
- Each system shares several common elements, including primary reliance on passive adverse event collection for marketed devices, but vary widely in their allocation of stakeholder responsibilities and mechanisms for evaluating the performance and safety of approved devices.
- Postmarket surveillance may be improved through greater system transparency, scheduled re-examination of approved devices, and balancing central and local control.

## Introduction

Medical devices play an increasingly vital role in health care delivery around the world. These technologies are defined in distinction to drugs as an “instrument, apparatus…machine…implant…or other similar or related article…which is…intended for use in the diagnosis of disease or other conditions, or in the cure, mitigation, treatment, or prevention of disease…and which does not achieve its primary intended purposes through chemical action” [1]. In recognition of the importance of medical devices, the World Health Organization established a Medical Device Unit to focus research and policy on prioritizing access to medical devices in low-resource settings, dessemination of innovations, and training of biomedical personnel to support the use of devices worldwide [2]. While medical devices offer opportunities for improved diagnosis and management of disease, they also can carry substantial risks. Governmental regulatory bodies considering new medical device approval balance the goals of expanding therapeutic options with safeguarding public health. Wherever the standard for market authorization is set, questions about a device's safety and effectiveness will remain after introduction into clinical practice. However, medical devices raise several unique challenges, including operator variability and procedural learning curves, permanent implantation, and the technological complexity of some devices.

After a new medical device is brought to market, the process of postmarket surveillance (PS) provides an ongoing assessment of safety and effectiveness. High-profile international public health crises involving widely used implantable cardioverter-defibrillator leads [3],[4], joint prostheses [5], and breast implants [6] raise questions regarding the strengths and weaknesses of different approaches to device PS worldwide. Though only limited quantitative measures of PS guide policy decisions [7], we evaluated the range of device PS strategies in four important medical device markets—the US, EU, Japan, and China. The US and EU represent large markets that are entertaining substantial reforms to PS practice. Japan and China are, respectively, mature and emerging international markets that have systems that could inform ongoing policy debates in the US and EU and be influenced by their practices in turn. Our goal was to identify “best practices” from among these countries that could support the public health goals of all device regulatory systems.

## United States

After a device is approved in the US, companies must maintain quality manufacturing control systems [8]. They must also report to the US Food and Drug Administration (FDA) adverse events brought to their attention by their employees or user facilities [9], providing patient demographic data, clinical information, and procedure details. Reports are collected in a publicly searchable database, though with variable content and quality. For example, most reports originate from manufacturer representatives, while health care providers have no mandate to report adverse events, and rarely do so [10].

The FDA may require manufacturers to conduct PS studies in two ways. First, “post-approval studies” may be appended to the approval of devices evaluated through the premarket approval (PMA) or Humanitarian Device Exemption (HDE) pathways, which include high-risk devices or those serving patients with rare diseases, (respectively), where premarket testing may be especially limited. Second, so-called “522 studies” (named in reference to the relevant legislation) for select devices (including those medium- or lower-risk devices cleared through the 510(k) pathway based on risk or other criteria) may also be required [11]. (In contrast to the PMA or HDE processes, the 510(k) process clears new devices based on a principle of “substantial equivalence” to a marketed device, indicating that the device at issue raises no new safety or effectiveness concerns. This process rarely requires new clinical data [12].) FDA posts the status of post-approval studies and 522 studies on public websites [13],[14].

When PS points to potential device problems, the FDA issues safety communications to inform patients and clinicians. Actual patient harms trigger safety alerts from the FDA, manufacturers, or distributors [15]; for example, a 2012 safety alert described a defective component in an automated external defibrillator that led to unexpected failure to deliver high-voltage therapy [16].

A recall reflects systemic concerns with a device. A manufacturer may conduct a recall on its own, or in response to an FDA request, and is responsible for developing the strategy for managing the recall process. More serious recalls, such as those issued for metal-on-metal designs for hip prostheses [17], invoke stricter FDA oversight, including follow-up and auditing of communication to providers or end-users. Publicly searchable databases track safety alert and recall information.

Recently, the FDA proposed adding a unique device identifier (UDI) system to its PS activities. UDIs allow linkage of specific devices to clinical information that can enhance the context of adverse event reports [18]. UDIs might facilitate rapid notification of devices' use and performance characteristics, support more accurate and timely aggreggation of adverse event data, and enable better coordiation of recalls [19].

## European Union

Devices are certified for marketing approval by private, for-profit Notified Bodies (NBs) around the EU based on adherence to European Commission directives that describe standards for device manufacturing, labeling, and expected performance and safety profiles. Competent Authorities (CAs) in each member state oversee NBs and have primary responsibility for PS; there is no equivalent to the FDA or the European Medicines Agency for devices in the EU. Directives also describe the basic standards for manufacturing quality-control systems and responsibilities for adverse event reporting. Non-binding European Commission guidance documents offer greater detail regarding handling adverse events, and communicating safety concerns. They also provide templates for data collection and reports, including “clinical evaluation reports,” which are intended to provide an outline of the technology underlying a specific device and current clinical data supporting its use, ideally in reference to established standards or similar devices [20]. In practice, each country variously interprets the requirements for quality assurance and adverse event reporting.

Manufacturers must report “any deterioration in the characteristics and performances of a device, as well as any inaccuracies in the instruction leaflet which might lead to or might have led to the death of a patient or to deterioration in his state of health” to CAs where the event occurs. A template outlines the required information to be sent to CAs. CAs must also have processes for collecting reports from manufacturers, relaying to manufacturers event reports submitted to CAs by users, and assessing ongoing risks associated with reported incidents or recalls. Member states notify other states if an assessment of device-related events leads to specific remedial measures.

CAs submit adverse event and recall data to the European Databank on Medical Devices (EUDAMED), a central but non-public database run by the European Commission. Some CAs maintain independent publicly available databases of device information. For example, the UK Medicine and Healthcare Products Regulatory Agency (MHRA) keeps adverse event and recall information in a searchable web portal.

European Commission directives do not grant authority to NBs or CAs to require post-approval studies. NBs as part of their review of individual devices can provide guidance for PS, though there is no evidence that studies or registry development are commonly (or even occasionally) required as conditions of approval. Neither the clinical data forming the basis for approved devices nor the existence, if any, of post-approval studies are systematically publicized because there is no requirement for NBs, manufacturers, or CAs to do so.

Manufacturers and regulators have obligations under the directives to manage adverse events and safety problems with marketed devices. Field Safety Corrective Actions (FSCAs) are “actions taken by manufacturers to reduce a risk of death or serious deterioration in the state of health associated with the use of a medical device” already on the market. These actions range from changes in labeling to withdrawal of products. Each CA must disseminate National Competent Authority Reports, which outline major safety issues for medical devices for the CAs of all member states.

Though not yet formalized into directives, recent European Commission suggestions for device PS reform include use of UDIs and connecting UDIs to EUDAMED. The provisions also include improved coordination among CAs and greater oversight of NBs, including clarification of NBs' responsibility and authority to conduct unannounced inspections of manufacturing facilities and audits of collected documentation related to adverse events.

## Japan

Device regulation in Japan is led centrally by two government agencies: the Pharmaceuticals and Medical Devices Agency (PMDA) and the Ministry of Health, Labor, and Welfare (MHLW). Under Japan's Pharmaceutical Affairs Law, MHLW has the authority to issue approvals for new devices and supervises PS including adverse event reporting and recalls. The PMDA provides the analytic work that informs MHLW's decisions, including inspections and premarket evaluations. PS in Japan includes systems for reporting foreign and domestic adverse events, identification of safety signals emerging from international markets, and postmarket studies [21]. Manufacturers are required to report adverse events directly to MHLW, and are the source of the overwhelming majority of these reports. Manufacturers must also track and report events that occur outside Japan for similar or related devices. Health care providers are required by law to make an effort to cooperate with manufacturers when they are actively investigating potential safety problems [22]. Analysis of reports by the PMDA may conclude that further investigation is required, or impose additional safety measures, such as changes in labeling. PMDA hosts a public database of adverse event and recall data available, as well as a database for updated package inserts [23].

PS studies may be required by PMDA for select devices. Approval of particularly risky devices may be paired with requirements to actively monitor domestic use of the device for up to five years, or for a pre-specified number of cases. Certain higher risk devices must “re-file” applications 3–7 years after initial marketing approval [24]. Sponsors aggregate information from health care providers, clinical trials, and published studies—such as foreign and domestic observational research or experiences from registries—to demonstrate that the device at issue is performing as intended and is providing the expected safety and effectiveness results. Theoretically, MHLW can withdraw marketing approval after a re-filing, though in practice this has not occurred.

Recalls arising from domestic incidents may include problems with documentation or reporting, as well as those related to adverse events. Foreign recalls do not automatically trigger a recall in Japan, but if the sponsor is a global company, marketing a device in Japan that has been recalled elsewhere is generally untenable.

## China

The central government's Ministry of Health (MOH) is responsible for drafting basic device oversight regulations and overseeing their implementation through the State Food and Drug Administration (SFDA) [25], which was recently elevated to a ministerial-level agency directly under the State Council and renamed the China Food and Drug Administration (CFDA) in an effort to reduce fragmentation and consolidate power [26]. Changes mostly affected food regulation, but its governmental promotion has granted drug and medical device authorities more regulatory capacity and ability to seek additional resources [27].

Provincial and municipal agencies serve as first-line responders when adverse events are reported and support the CFDA in monitoring and taking action at the regional level [28]. The CFDA is responsible for collecting, aggregating, and analyzing adverse event data from across all provinces and regions. Manufacturers, distributors, and users of medical devices must inform regional monitoring institutions of death- and injury-related adverse events [29]. Most cases appear to be reported by medical institutions [30]. The CFDA hosts a central but non-public online database that tracks adverse medical events.

Product approvals are renewed quadrennially, using data gathered since the initial registration. Regulations were recently drafted to simplify this re-registration process by only requiring new documentation to establish effectiveness and safety of significant changes made in relevant products. Device vigilance reports consolidating and analyzing adverse events related to these products must also accompany re-registration [31]. Post-approval studies are required for certain drug groups [32], but the CFDA has yet to set similar requirements for medical devices. However, manufacturers of newer imported medical devices have reportedly been compiling outcomes data from routine clinical experience, such as that with newer generations of coronary stents [33].

Device recalls are initiated by manufacturers based on self-investigation and assessment of product defects, ranging from eliminating defects through relabeling or software upgrades, to full market withdrawal [34]. The manufacturer regularly updates regional authorities on the recall status, and submits a summary report after completion. For a medical device that has caused severe injuries or death, regional authorities and the CFDA organize groups of representatives and experts from device monitoring institutions, manufacturers, and scientific research teams to determine whether to revoke the registration certificate. Manufacturers and companies are restricted from securing regulatory approvals or licenses for 2 years if they are involved in manufacturing counterfeit or substandard products, or if they cause serious safety events due to violation of drug/device laws [35].

China's regional structure grants substantial autonomy to provincial health departments. For example, Shanghai has developed a traceability program for implantable medical devices, adopting a UDI system linking implantable medical devices directly to patients across over 100 hospitals. The database provides detailed records of adverse events and patients involved, allowing the CFDA to hold back potentially dangerous products while still in inventory and limit future injuries [36].

## Best Practices

These four PS systems share several features (Table 1), including primary reliance on passive adverse event collection for marketed devices. At a broad level, these systems allocate responsibility among stakeholders differently, with heavier burdens on manufacturers in Japan and China contrasting with significant authority devolved to for-profit third-parties in the EU. The US may lie between these extremes, with the FDA mandate to protect public health buttressed by obligations on industry. To date, no empirical studies adequately characterize one system as superior to another. However, we argue that this comparative analysis suggests three best practices with promise to promote public health.

**Table 1:** Key features of four device postmarket surveillance systems.

System Feature	US	EU	Japan	China
**Transparency**	Premarket data and PS study status for high-risk devices publicly available. Public databases for reported adverse events and recalls.	Basis for device approval and any postmarketing commitments largely unknown. EU-wide adverse event data not accessible, though individual countries post PS events in non-systematic manner	Public posting of approvals, adverse event data, and notices	Public website listing all approved devices including labeling, but clinical data and collected adverse event data not public
**Formal re-examination**	Product performance reports may be submitted from PS studies registries, but no formal process for renewing approval for specific indications	Clinical evaluation reports summarize PS data but are not consistently produced and are not used to evaluate renewal of CE marking	Statutorily-required formal re-examination period for selected devices	Statutorily-required formal re-examination period for selected devices
**Central versus local control**	Device regulation centralized at FDA; individual states may not impose stricter standards on manufacturers for marketing	EU provides guidance but directives are interpreted by national Competent Authorities and private Notified Bodies	Centralized process organized by Ministry of Health, Labor and Welfare and its Pharmaceutics and Medical Devices Agency	Central CFDA provides oversight for provincial authorities, however opportunities for local initiatives exist

First, we found substantial variation in public access to PS processes and data. On one end of this spectrum, the US FDA provides access to many high-risk PS decisions through advisory panel meetings and public “conditions of approval” for select devices that include the design and rationale of PS studies. For example, a 1,600-patient post-approval study was mandated for a novel subcutaneous defibrillator system to address specific concerns including shock effectiveness and discrimination of arrhythmias [37],[38]. As this device is permanently implanted and treats a life-threatening problem (ventricular arrhythmia) for which an established alternative (transvenous defibrillators) exists, a relatively cautious assessment of the new technology was justified. These studies and their status are available on an FDA website [39], though it remains unclear how the content of these studies will be released publicly.

The EU approach to the subcutaneous ICD demonstrates the other end of the transparency spectrum. This device was marketed in 2009 after a 55-patient study demonstrating that the device worked as intended [45]. It is not publicly known which NB evaluated the device, the data supporting its evaluation, and the nature of PS plans, if any. (This device is not yet approved for marketing in Japan or China.) Since then, PS data have emerged non-systematically from published accounts of real-world clinical experiences in Holland [40] and Germany [41] totaling 187 patients. Though the newly created clinical evaluation reports should provide updates to marketed devices' technical files, these reports are not made public and their consistency, rigor, and utility is not known. Indeed, a separate industry has arisen to prepare and submit clinical evaluation reports. As one website reassures prospective clients, “If conducting a well-designed report seems daunting and time-consuming, especially with Notified Bodies demanding updates on commercial devices, let [us] help you.” [42]

Physicians, device safety researchers, payers, and policymakers should be able to access data supporting approval of new devices, particularly high-risk devices, to understand uncertainties related to safety and effectiveness and the specific goals of PS. Public accountability can also help ensure that post-approval studies are completed in a timely manner and published in peer-reviewed venues or through the regulatory authority itself.

Second, the requirement in Japan (and evolving in China) that select devices re-file with the regulatory authority after a fixed time period offers another powerful PS tool. Scheduled reassessment of accumulated clinical experience allows regulators to assess whether safety and effectiveness expectations have been met. Reexamination supports review of labeled indications or safety concerns, and also provides an avenue for evaluating whether, in retrospect, the premarket process for a specific device adequately anticipated safety and effectiveness concerns. This knowledge can provide feedback for that device's premarket review and inform future evaluation of similar devices by influencing study design and sample size calculations affecting safety concerns.

An interesting case study of this approach to PS paradoxically comes from the US, where in 2006, the FDA convened an advisory panel to evaluate current knowledge on the safety and effectiveness for the two models of widely used drug-eluting coronary stents [43]. This assessment followed emerging concerns that these stents exposed patients to previously unknown risks for late stent thrombosis, a rare but catastrophic event. This formal evaluation—held about 3 years after approval of these devices—contributed to international momentum for endpoint definitions applied to future stent evaluation and PS. A scheduled re-examination of novel ICD lead models—*before* major recalls—perhaps would have helped identify safety problems more quickly, or at least provided more structured PS guidance. To our knowledge, no device in Japan or China has been removed from the market at the time of its re-examination. Yet this may reflect a strength of this approach, as manufacturers faced with a strict deadline with meaningful consequences are strongly motivated to address post-market concerns well in advance of the re-examination. Looking forward, we argue that a scheduled re-examination for devices such as transcatheter aortic valves, left ventricular assist devices, or new hip prostheses would support PS for these new therapies, and may motivate completion of post-approval requirements.

Novel, important technology needs to be made accessible to patients, but it is sensible to pair approval of select high-risk devices with comprehensive, public re-assessments of available safety and effectiveness data at a predetermined interval. Such meetings are likely to be most effective when paired with statutorily defined enforcement options, including re-labeling, further study, or withdrawal. These re-examinations hold manufacturers and regulators accountable for their decisions and subjects pre-approval estimates of device performance to scrutiny.

Third, the systems we reviewed strikingly different balance between central and regional control. The US system is tightly centralized, so much so that states have limited authority to impose additional requirements for manufacturers [44]. By contrast, EU directives set broad parameters, but assign responsibility to CAs and NBs with loose coordination and supervision. Perhaps as a result, competition between NBs tends to focus on speed and simplicity for manufacturer clients. Resting between these extremes, China's centralized system maintains strong central oversight while granting some autonomy to provincial CFDA agencies. This supports the role of provincial health departments as the first line of response to adverse medical device events, yet allows for relatively straightforward pooling of national-level data. The existence of regional autonomy within a centralized structure may create faster responses to adverse events and make implementing policy changes easier, and may be worthy of exploration in both the US and EU.

## Conclusion

Even the most rigorous clinical testing of experimental devices will leave some safety and effectiveness questions unanswered. At the same time, broader dissemination of new technology and longer clinical experience may identify unforeseen concerns. For example, many health policy discussions around improving PS have urged regulators to supplement passive collection of adverse events with ongoing, dynamic assessment of safety and effectiveness during the full life cycle of marketed devices through mechanisms such as UDI systems. However, these strategies will take time to design and implement. As device PS systems move towards more active surveillance, our review reveals important features of current systems around the world that promote coordination among regulators, manufacturers, clinicians, and patients. Broader use of these strategies could preserve patients' access to new technologies while protecting them as well as possible from devices that later turn out to be unsafe or ineffective.

## Abbreviations

CA: Competent Authority
EUDAMED: European Databank on Medical Devices
FDA: US food and Drug Administration
FSCA: Field Safety Corrective Action
HDE: Humanitarian Device Exemption
MHLW: Japan Ministry of Health, Labor, and Welfare
MHRA: UK Medicine and Healthcare Products Regulatory Agency
MOH: China Ministry of Health
NVB: Notified Body
PMA: premarket approval
PMDA: Japan Pharmaceuticals and Medical Devices Agency
PS: postmarket surveillance
SFDA: China State Food and Drug Administration
UDI: unique device identifier
